# Supplementary material for: A System for Standardizing and Combining U.S. Environmental Protection Agency Emissions and Waste Inventory Data
Source: Appl Sci (Basel). Author manuscript; Available in PMC 2022 Jun 8. (PMC9175305; doi:10.3390/app12073447)
Supplement: Supplementary Material [file NIHMS1808810-supplement-Supplementary_Material.pdf]

Supplementary Information for A System for Standardizing and Combining U.S. Environmental Protection Agency Emissions and Waste Inventory Data

Ben Young, Eastern Research Group, Inc.

Wesley W. Ingwersen*, U.S. Environmental Protection Agency, Office of Research and Development

Matthew Bergmann, GDIT and U.S. Environmental Protection Agency, Office of Research and Development

Jose D. Hernandez-Betancur, Oak Ridge Institute for Science and Education, hosted by U.S. Environmental Protection Agency, Office of Research and Development

Tapajyoti Ghosh, Eastern Research Group, Inc.

Eric Bell, Eastern Research Group, Inc.

Sarah Cashman, Eastern Research Group, Inc.

National Emissions Inventory Reporting Thresholds

The U.S. Environmental Proctection Agency (USEPA) has national air quality standards for six criteria air pollutants (CAPs) (NO_2, O_3, SO_2, PM, CO, and Pb). Facilities can either directly emit some of these pollutants, or the pollutants may be formed through atmospheric reactions (i.e., O_3, PM). The National Emissions Inventory (NEI) was established to track and help achieve these CAPs National Ambient Air Quality Standards (NAAQS). Type A sources (i.e., large sources) must report to NEI every year, while Type B sources must report to NEI every three years. The emission thresholds defining Type A, Type B, and sources in nonattainment areas are listed in Table S1 (from 40 CFR §51, Appendix A, Table 1). Nonattainment areas are those that do not meet the NAAQS. These thresholds, with the exception of lead, are based on the potential to emit rather than the actual amount emitted in a year. The potential to emit represents the highest amount of certain pollutants that a business could potentially release into the air regardless of whether that release quantity occurs. The potential to release is largely based on information regarding equipment design and controls in place. If a facility qualifies as a Type A or B source for any of the six CAPs, it must report all CAPs. These thresholds just determine if a facility should report to NEI under a specific category, and do not represent the actual flows provided in NEI. The facility will report the actual release values. The actual release value, however, should be used as the threshold to report lead emissions. Note that the AERR thresholds were revised to reflect 70 tons/year for PM10, PM2.5, and PM precursors (i.e., SO_2, VOC, NO_X, PM2.5, and NH_3) for sources within PM10 and PM2.5 nonattainment areas for years after the 2014 NEI. The smaller sources that are not included in the point source inventory are intended to be

included in the nonpoint inventory. Thresholds are not available for HAPs, as these are voluntary fields in NEI.

Table S1: Emissions Thresholds in Tons per Year for Type A Sources, Type B Sources, and Sources in Nonattainment areas. From: 40 CFR §51, Appendix A, Table 1.

Pollutant	Emission Threshold (tons per year of potential to emit)		
	Every Year	Triennial	
	Type A sources	Type B sources	NAA sources
Sulfur Dioxide (SO_2)	≥2500	≥100	≥100
			$PM_{2.5}$ (serious) ≥70
Volatile Organic Compound (VOC)	≥250	≥100	≥100
			O_3 (serious) ≥50
			O_3 (severe) ≥25
			O_3 (extreme) ≥10
			$PM_{2.5}$ (serious) ≥70
Nitrogen Oxides (NO_x)	≥2500	≥100	≥100
			$PM_{2.5}$ (serious) ≥70
Carbon Monoxide (CO)	≥2500	≥1000	O_3 (all areas) ≥100
			CO (all areas) ≥100
Lead (Pb)		≥0.5 (actual)	≥0.5 (actual)
Primary Particulate Matter < 10 µm (PM_{10})	≥250	≥100	≥100
			PM_{10} (serious) ≥70
Primary Particulate Matter < 2.5 µm ($PM_{2.5}$)	≥250	≥100	≥100
			$PM_{2.5}$ (serious) ≥70
Ammonia (NH_3)	≥250	≥100	≥100
			$PM_{2.5}$ (serious) ≥70

Table S2: Percent of facilities that report Hazardous Air Pollutants by method. S/L/T: State, local, or tribal agency.

Method	2011	2014	2017
Reported by S/L/T and Augmented by EPA	26%	22%	22%
Reported by S/L/T and no EPA Augmentation	36%	37%	37%
Augmented by EPA with no S/L/T Reported	38%	41%	41%
